# Multicenter Evaluation of Frequency and Impact of Activity Infiltration in PET Imaging, Including Microscale Modeling of Skin-Absorbed Dose

**DOI:** 10.2967/jnumed.123.265891

**Published:** 2023-07

**Authors:** John J. Sunderland, Stephen A. Graves, Dusty M. York, Christine A. Mundt, Twyla B. Bartel

**Affiliations:** 1 Department of Radiology, University of Iowa, Iowa City, Iowa;; 2 Chattanooga State Community College, Chattanooga, Tennessee; and; 3 Global Advanced Imaging, Little Rock, Arkansas

**Keywords:** infiltration, extravasation, dosimetry, Monte Carlo

## Abstract

There has been significant recent interest in understanding both the frequency of nuclear medicine injection infiltration and the potential for negative impact, including skin injury. However, no large-scale study has yet correlated visualized injection site activity with actual activity measurement of an infiltrate. Additionally, current skin dosimetry approaches lack sufficient detail to account for critical factors that impact the dose to the radiosensitive epidermis. **Methods:** From 10 imaging sites, 1,000 PET/CT patient studies were retrospectively collected. At each site, consecutive patients with the injection site in the field of view were used. The radiopharmaceutical, injected activity, time of injection and imaging, injection site, and injection method were recorded. Net injection site activity was calculated from volumes of interest. Monte Carlo image–based absorbed dose calculations were performed using the actual geometry from a patient with a minor infiltration. The simulation model used an activity distribution in the skin microanatomy based on known properties of subcutaneous fat, dermis, and epidermis. Simulations using several subcutaneous fat-to-dermis concentration ratios were performed. Absorbed dose to the epidermis, dermis, and fat were calculated along with relative γ- and β-contributions, and these findings were extrapolated to a hypothetical worst-case (470 MBq) full-injection infiltration. **Results:** Only 6 of 1,000 patients had activity at the injection site in excess of 370 kBq (10 μCi), with no activities greater than 1.7 MBq (45 μCi). In 460 of 1,000 patients, activity at the injection site was clearly visualized. However, quantitative assessment of activities averaged only 34 kBq (0.9 μCi), representing 0.008% of the injected activity. Calculations for the extrapolated 470-MBq infiltration resulted in a hypothetical absorbed dose to the epidermis of below 1 Gy, a factor of 2 lower than what is required for deterministic skin reactions. Analysis of the dose distribution demonstrates that the dermis acts as a β-shield for the radiation-sensitive epidermis. Dermal shielding is highly effective for low-energy ^18^F positrons but less so with the higher-energy positrons of ^68^Ga. **Conclusion:** When quantitative activity measurement criteria are used rather than visual, the frequency of PET infiltration appears substantially below frequencies previously published. Shallow doses to the epidermis from infiltration events are also likely substantially lower than previously reported because of absorption of β-particles in the dermis.

## Visual Abstract

**Figure:**
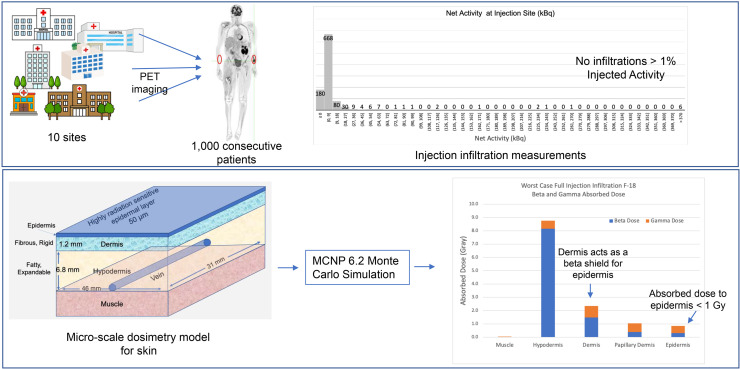

There has been recent focus within the nuclear medicine community on the frequency and clinical consequences associated with injected activity infiltration in both diagnostic imaging and therapeutic applications. The situation is complicated by the variety of radionuclides in clinical use, the range of injected activities, their different half-lives, and their different radiation emissions, all of which affect the potential adverse impact of infiltration. The dearth of reported acute adverse events associated with activity infiltration over the last 50 y, spanning tens of millions of nuclear medicine injections, makes it difficult to draw systematic conclusions on cause and frequency. This is further complicated by an incomplete understanding of tissue-specific radiation dose effects, a lack of consistent definition of what is considered a consequential infiltration event, and the unclear impact of consequential infiltration on clinical care.

Relatively recently, several investigations into the frequency and severity of infiltration events in the nuclear medicine space have been published, including a relatively complete systematic review covering both diagnostic and therapeutic events (1). Several articles have been published on infiltrations in ^18^F-FDG PET imaging (2*,*^3^). There are additional publications that report the dosimetric implications of infiltrated activity (4*,*^5^).

Infiltration can adversely impact a patient in 3 ways. The first is the safety issue associated with potential tissue damage due to local absorbed radiation dose. The second is the possibility that a suboptimal injection may negatively impact the quality of the scan, making it less diagnostic. The third is that a suboptimal injection resulting in infiltration will impact image quantitation and result in the potential for misinterpretation, particularly in the case of response to therapy. In all cases, understanding the frequency and severity of infiltration events will help inform recommended protocols.

In this work, we performed a controlled, retrospective, quantitative multicenter study of dose infiltration involving 10 imaging sites and 1,000 patients using PET imaging. The choice of PET was primarily due to its absolute quantitative nature and its superior sensitivity and resolution, providing the ability to quantitate the radioactivity at the injection site and estimate the infiltrate tissue mass at the imaging time point. Further, to better understand the potential for biologic impact on the patient, a patient-specific case of suboptimal injections was analyzed using imaging data and patient-specific Monte Carlo dose calculations accounting for microscale anatomy of the skin to understand the respective dosimetric implications on the epidermis, dermis, and subcutaneous soft tissue. To our knowledge, dose estimates taking into account specific infiltration geometries and the impact of tissue radiosensitivity have not previously been reported. The dosimetric approach presented here can be generalized and may provide a valuable contextual approach to dose estimation in other nuclear medicine applications.

Critical to the dosimetric context of radiopharmaceutical infiltration is an understanding of the macroanatomy and microanatomy of the skin, as well as their respective thicknesses and associated fluid dynamics. The highly proliferative and radiation-sensitive epidermis, with a thickness of 50–70 μm, is the dose-critical structure (6). Beneath the epidermis are the largely acellular papillary dermis (∼15 μm) and reticular dermis (∼1,000 μm) (7). The papillary dermis and reticular dermis consist primarily of dense, fibrous connective tissue with little volumetric expansion capability. Beneath the dermis lies the subcutaneous fat layer, with a small proportion of structural connective tissue. Subcutaneous tissue is lightly cellular and highly deformable. Its thickness increases with patient fat content, varying between 1 and 10 mm or more. Veins with diameters large enough for cannulization lie at the base of the subcutaneous tissue and are just superior to muscle fascia. The skin anatomy and dimensions used are illustrated in Figure 1. Monte Carlo simulations performed in this work, accounting for the anatomic distribution of infiltrating activity on absorbed dose to different skin components, should provide a deeper understanding of the potential for deterministic radiation effects after radiopharmaceutical infiltration.

**FIGURE 1.**
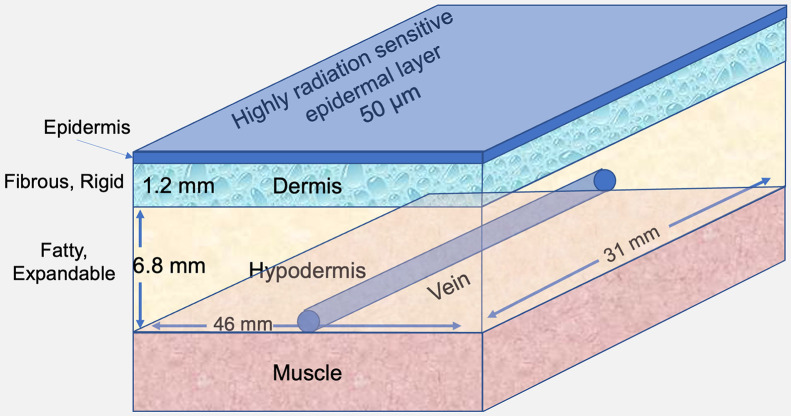
Schematic of skin anatomy with primary dimensions used in Monte Carlo absorbed dose calculations.

We hypothesize that the quantitative analysis of this large and diverse PET imaging patient cohort will better define the frequency and clinical impact of infiltrations, specifically as relates to the 3 adverse event categories (tissue damage, diminished diagnostic image quality, and quantitative accuracy). We further hypothesize that Monte Carlo–based absorbed dose calculations using patient-specific infiltration activities and geometry will better inform potential tissue damage models both in PET and, by inference, in other nuclear medicine applications.

## MATERIALS AND METHODS

### Patient Population

In total, 1,000 PET/CT oncology patient studies were collected from 10 different imaging institutions. To avoid bias associated with a single imaging model, a variety of institutions was chosen, including 1 academic medical center, 2 private radiology groups, 2 private oncology groups, 1 community hospital, 2 multispecialty groups, and 1 research facility. Approximately 50 patients from smaller clinics were combined into a tenth “institution.” This diverse group of institutions should represent the variety of injection skills and injection techniques typically used in the clinical PET environment. At each of the institutions, consecutive patients who had the injection site in the field of view were analyzed. To avoid overrepresentation from a single site, no site contributed more than 230 patients (minimum, 37; maximum, 230; median, 74). Several radiopharmaceuticals were represented in the patient cohort, including ^18^F-FDG, ^18^F-fluciclovine, ^18^F-DCFPyL, ^68^Ga-DOTATOC, and ^68^Ga-DOTATATE. All injection methods were allowed, including intravenous cannulation, butterfly injection, direct stick, port access, and peripheral insertion of a central catheter line. The informed consent requirement for this retrospective analysis was waived by the institutional review boards or equivalent authorities.

### Imaging Data

For each patient, the following data were collected to quantitatively analyze any infiltration: radiopharmaceutical, net injected activity (Bq), time of injection, time of imaging, injection site (right or left antecubital, forearm, wrist, or hand), and injection type (e.g., intravenous catheter, port, or peripherally inserted central catheter). PET/CT images were analyzed by 1 of 2 investigators. Volumes of interest were drawn around the injection site to include all potential activity associated with the injection. A similar volume of interest of nearly equivalent volume was drawn on the contralateral side.

### Injection Site Analysis

Each reader subjectively assessed whether the injection site was clearly visualized, barely visible, or not visible. Mean and maximum radioactivity concentration (Bq/cm^3^) and volume of interest (cm^3^) were measured at the injection site. Net activity at the injection site was calculated as the difference between activity at the injection site ([mean radioactivity concentration] × [volume of interest]) and activity on the contralateral side. The net percentage injected activity at the injection site was calculated.

A measured activity of more than 370 kBq (10 μCi) at the injection site, decay-corrected back to time of injection, was used as the threshold for further analysis of the clinical impact of infiltration. On average, this threshold represented approximately 0.1% of the average injected dose of 407 MBq (11 mCi).

### Patient-Specific Dosimetry

Presented in this work is a single-sample, patient-specific skin dose calculation performed using the image-based geometry of a minor infiltration event (as no major events were found). Monte Carlo simulation was performed using MCNP, version 6.2. For each radioactive emission mode (β-particles, discrete electrons, and photons), 1 × 10^7^ emission events were simulated, thereby providing less than 1% estimated statistical uncertainty in absorbed dose results in each skin layer for each emission type (excluding uncertainty from discrete electrons beyond their practical range). Static geometry was assumed. A subcutaneous tissue thickness of 6.8 mm (patient-specific based on CT images) and a dermal thickness of 1.2 mm were simulated. An effective clearance half-life of 30 min was modeled for the infiltrated activity based on the literature (4). For ^68^Ga simulations, 25 min was used. The Monte Carlo infiltration dimensions were taken from the patient’s CT scan and were 3.6 × 2.1 cm in *x* and *y,* with *z* being 1.2 mm in the dermis and 6.8 mm in the subcutaneous tissues. Because the exact equilibrium distribution of the infiltrated activity into the expandable subcutaneous tissue and the fibrous dermal tissue is unknown, multiple simulations were performed. The actual equilibrium distribution is currently under study, and preliminary experiments suggest an approximately 10:1 hypodermal-to-dermal concentration ratio. However, to be conservative, 10:1, 5:1, and 2:1 ratios were simulated. Dose contributions from β-particles, photons, and electrons were separately estimated from the MCNP model.

## RESULTS

### Frequency and Severity of Infiltrations

Injected activities averaged 405 ± 78 MBq from all sites, with 952 of the injections being ^18^F-FDG and the others distributed among other approved radiopharmaceuticals. The distribution of injection sites was approximately two-thirds (645 patients) antecubital injections, with 135 hand injections, 111 forearm injections, and 34 wrist injections. All venous access, when not performed through a peripherally inserted central catheter line (3 patients) or port access (66 patients), was performed using venous cannulation, with no reported direct sticks or butterfly access.

**FIGURE 2.**
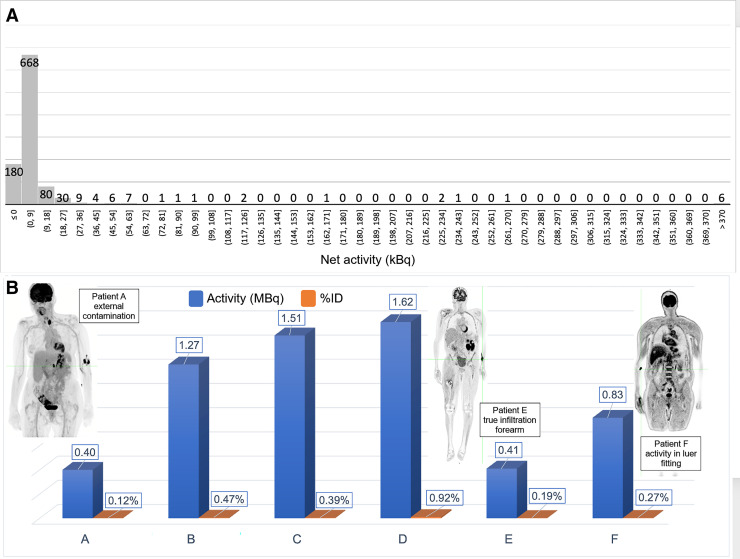
(A) Histogram distribution of net activity (MBq) at injection site. (B) Detail of 6 infiltrations above 0.4 MBq (10 μCi) with percentage injected activity. Patient E’s injection geometry was used in Monte Carlo simulation.

The distribution of net activity measured at the injection site is shown in Figure 2A. In 985 of the 1,000 patients, there was less than 74 kBq (2 μCi) at the injection site. Of the 15 patients with higher activities, none exceeded 1.65 MBq (44 μCi), with no injection site receiving more than 1% of the net injected activity. Details on the 6 patients who had more than 370 kBq (10 μCi) at the injection site are shown in Figure 2B. Interestingly, of the 6 patients with more than 370 kBq at the injection site, only 3 were infiltrations whereas the others were either external contamination or activity trapped in external tubing or fittings.

### Probability of Infiltration

If we assume that data from the 10 sites were a representative sample of PET injection techniques and we use an overly conservative definition that activity of less than 1% of the injected dose at the injection site is not a clinically meaningful infiltration event (no clinically negative impact), then we will have observed 0 infiltration events in our 1,000 patient sample. This information allows us to calculate a statistical 95% CI for the probability of an infiltration of more than 1%. When a simple binomial distribution calculation with a sample size of 1,000 is used, the 95% CI for an infiltration of more than 1% is 0–0.0037. Simply stated, with 95% confidence we can say that the rate for PET infiltrations of more than 1% of injected activity is between 0.00% and 0.37%.

### Dosimetry Results

**FIGURE 3.**
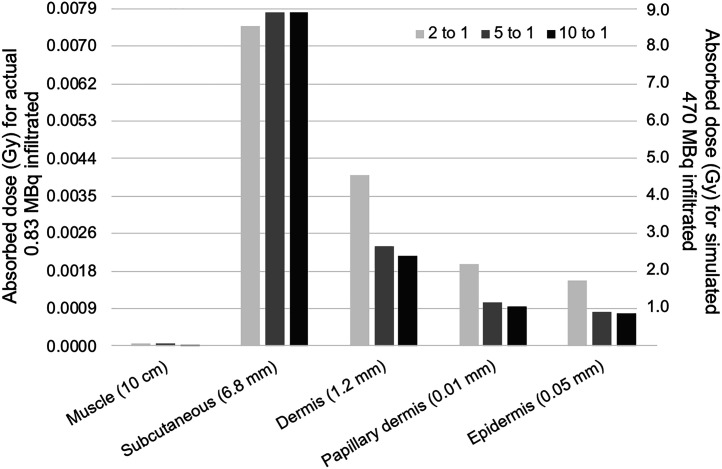
Absorbed dose for actual 0.41 MBq infiltrated (left *y*-axis scale) and for hypothetical full injection infiltration of 470 MBq (right *y*-axis scale) with 1.2-mm dermis.

Monte Carlo–generated absorbed radiation doses for the measured 0.41 MBq of ^18^F-FDG infiltrated for patient E (left *y*-axis) and for an extrapolated full-injection infiltration of 470 MBq (right *y*-axis) are shown in Figure 3. For the actual infiltration, absorbed doses were predictably highest in the expandable hypodermis, where most of the activity pools and delivers most of the positron energy deposition. Regardless of assumed equilibrium concentration ratios between subcutaneous and dermal tissue, absorbed doses narrowly ranged from 7 to 8 mGy. Absorbed dose in the dermis, at roughly 2 to 4 mGy, was generally more than 2 times lower than that in the subcutaneous tissue; however, at lower subcutaneous-to-dermal equilibrium concentration ratios, the dermal dose was substantially higher because of the larger proportion of the infiltrate resident in dermal tissue. The radiation-sensitive epidermal layer was estimated to receive between 1.5 and 4.2 mGy, depending on equilibrium concentration ratios. These absorbed radiation doses to the epidermis are roughly 1,000 times below the lowest absorbed doses at which even the most minor deterministic tissue injuries are reported (8*,*^9^). Given the small infiltrated activity, these results are not unexpected.

Extrapolating to a hypothetical 470-MBq infiltration, absorbed radiation doses to the radiation-resistant subcutaneous tissue range between 8 and 9 Gy, whereas the dose to the epidermis generally remains below 2 Gy, the lowest absorbed doses at which minor erythema has been seen in the most sensitive patients (reported from external-beam radiation therapy and fluoroscopy dose estimates (8*,*^9^)).

### β- and γ-Dose Distribution

**FIGURE 4.**
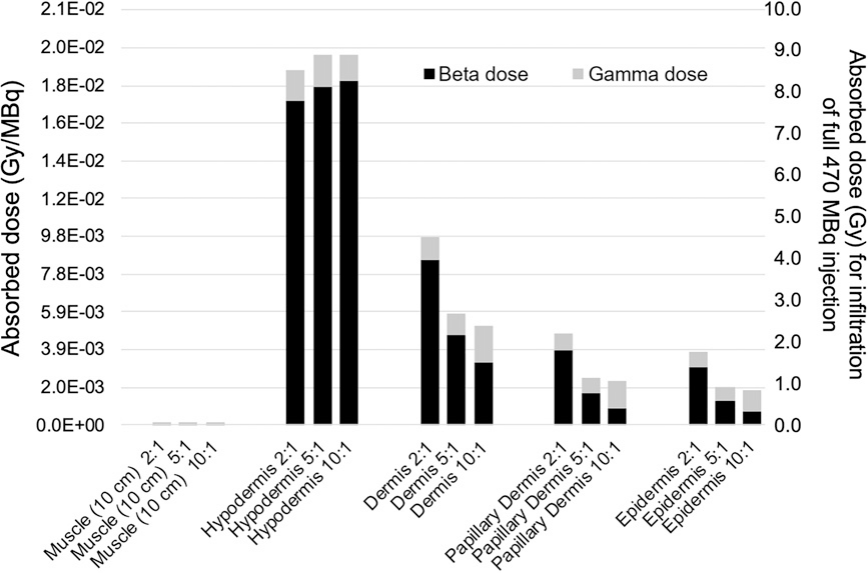
Absorbed dose per megabecquerel infiltrated for γ- and β-components for ^18^F in 5 tissue compartments (left *y*-axis) and assuming full 470-MBq activity infiltration (right *y*-axis).

The relative absorbed dose contributions of positron, γ-particle, and electron emissions were separately tabulated during the Monte Carlo simulation. Figure 4 breaks down the absorbed radiation dose per infiltrated activity (Gy/MBq) into its respective β- and γ-components for each of the 5 skin tissues simulated using the 1.2-mm dermal thickness and also presents estimated doses for the extrapolated 470-MBq infiltration case (right *y*-axis). It is important to note that the mean range of ^18^F positrons is approximately 0.6 mm, with only a very small fraction traveling greater than 1.2 mm, the thickness of the dermis. Also of note is the fraction of absorbed dose in the epidermal layer attributable to β-particles (positrons) relative to γ-particles for the different equilibrium concentration ratios. When the concentration in the dermal layer is low, the dermis only minimally contributes β-dose to the epidermis because of significant self-absorption, while simultaneously acting as an effective β-shield for positrons sourced in the subcutaneous tissue. Doses to other less radiosensitive tissue remain below 10 Gy for all circumstances simulated. This is likely about 5 times below the threshold for tissue damage within these largely acellular skin subanatomies.

### Impact of β-Endpoint Energy

Most infiltrated activity resides within the fractures between fat cells in subcutaneous tissue. β-penetration from the subcutaneous tissue through the dermis has the potential to generate a β-particle absorbed dose into the epidermal tissue only when the endpoint β-range of the infiltrate’s β-emissions is substantially longer than the dermal thickness, which is not the case with ^18^F. However, because ^68^Ga has a mean positron range of more than 3 mm, we simulated radiation doses to skin anatomies using patient A (Fig. 2) geometries with ^68^Ga as a source radionuclide to determine the potential impact of β-energy on skin injury.

**FIGURE 5.**
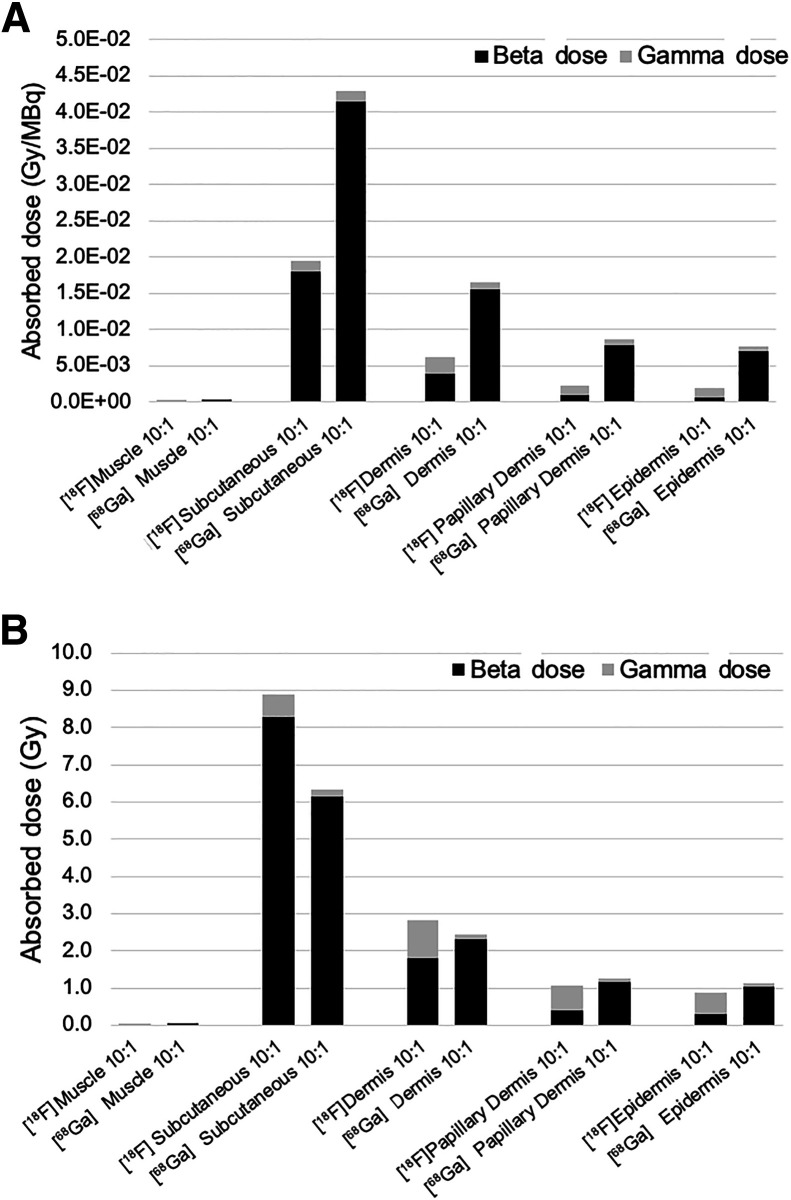
(A) γ- and β-contributions to absorbed dose for each subanatomy per megabecquerel infiltrated for ^18^F and ^68^Ga. (B) Relative γ- and β-contributions to absorbed dose for full infiltration of ^18^F and ^68^Ga.

Results comparing the absorbed dose of ^18^F with that of ^68^Ga on a per-decay basis are shown in Figure 5A. ^68^Ga predictably manifested an increase in absorbed dose per decay in all tissues, including, most significantly, the epidermis. Absorbed doses associated with a worst-case scenario of a 470-MBq ^18^F-FDG infiltration versus a 148-MBq infiltration of ^68^Ga (a complete activity infiltration) are shown in Figure 5B. There are substantial increases in both proportional and absolute β-dose to tissues, up to and including the epidermis, when the dermis is no longer an effective β-shield. Even at an injected activity of less than a third of its ^18^F counterpart, the total absorbed dose in the epidermis from a 148-MBq ^68^Ga infiltration is higher than for the 470-MBq infiltrated ^18^F activity.

## DISCUSSION

To date, studies investigating the frequency of nuclear medicine activity infiltration have not reported the quantitative activities of the infiltrations (3*,*^10^), which would seem to be a crucial variable. Additionally, those studies investigating the risks associated with nuclear medicine infiltrations have been based on simplified models that account for neither a realistic injectate distribution within the anatomic structures of the skin nor the relative radiation sensitivity of the different layers of the skin. This work is the first attempt, to our knowledge, to address these issues systematically.

### Frequency of Infiltration

With regard to the frequency and severity of infiltration, there are widely varying estimates reported in the literature. Osman et al. reported a relatively frequent 10% incidence of infiltrations in PET studies. These data, published in 2011, used exclusively 23- to 25-gauge butterfly needles for injection (3) rather than the currently recommended standard cannulation procedure for PET studies. In that study, an administration was considered extravasated if any remaining activity was visible at the injection site. In a smaller study in 2017, similar infiltration rates were identified with similar visually based criteria (11). Neither of these reports provided quantitative measurements of activity infiltrated despite the availability of these data through PET images. In our study, activity at the injection site was measured in 1,000 consecutive cases. It quickly became clear that simple visualization of activity was not evidence of meaningful infiltration, nor was it indicative of a poor, ineffective, or potentially dangerous injection. In fact, clearly visualized activity at the injection site was present in 460 of the 1,000 subjects but contained an average of only 34 kBq (0.9 μCi), which represents 0.008% of the net injected activity. We would argue that this is not representative of an actual infiltration event, which strongly suggests that visualization alone is not a meaningful criterion for infiltration and that the remarkably high sensitivity of PET imaging coupled with low background in peripheral tissues can lead to visually deceiving conclusions. Another important observation was that the activity near the injection site seen on PET was not necessarily anatomically within the patient. It is important to localize the activity on the CT portion of PET because several times the activity was actually external to the patient.

It is also important to note that venous cannulation was uniformly implemented at all 10 sites in this study and that with this practice, there was not a single clinically meaningful infiltration event observed in the 1,000 cases reviewed and analyzed.

### Skin Anatomy and Fluid Dynamics

The previous literature investigating radiation dose to the skin from infiltration events has largely ignored skin anatomy and the implications of fluid behavior in these tissues. In this work we consider both, as they would seem to have potentially significant implications in any dosimetric model.

**FIGURE 6.**
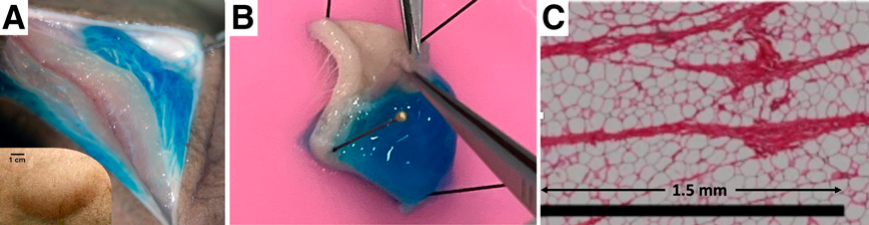
(A) Tumescent injection of 10 mL of saline (dyed blue) into porcine subcutaneous fat. Slicing into it reveals that liquid is held in subcutaneous tissue, forced to expand to accommodate in 5-cm diameter bleb (lower left insert). Lack of migration into dermis is seen. (Reprinted with permission of (12).) (B) ^18^F-FDG with blue dye subcutaneously injected into euthanized pig, excised 15 min after injection. ^18^F-FDG remains virtually exclusively in fat and does not appear to diffuse into dermis. (C) Histologic image of porcine adipose tissue. Septa are seen as magenta stripes. Scale bar is 1.5 mm. (Reprinted with permission of (14).)

Skin is a large and complex organ, but for our purposes it is critical to at least appreciate the structure, function, and dimensions of the major layers. The behavior of fluids when purposely or accidentally injected into the subcutaneous space has been abundantly reported in the literature (12–14), as tumescent fluid injections into the subcutaneous tissue for purposes of local anesthesia are common for several dermatologic procedures, including liposuction, cutaneous surgery, and drug administration. On injection, the fluid, under pressure, is trapped within the intracellular matrix of the subcutaneous tissue, as the fatty tissue expands severalfold. The fluid seeks and opens channels between adipose cells, as the fluid finds paths of least resistance (Fig. 6) (15). The initial local distribution occurs over the first few minutes, whereas subsequent absorption and dispersion via the circulation and lymphatic system occur over several hours, as has been observed and documented in nuclear infiltration studies by Osborne et al. (4). Insight from the literature informed our modeling assumptions regarding the distribution of concentrations between the subcutaneous tissue and the dermis. Figure 6A (12) demonstrates how 10 mL of stained saline injected tumescently into the subcutaneous tissue distributes but only minimally invades the dermis, if at all. A similar tumescent injection experiment was repeated by the authors using ^18^F-FDG in euthanized piglets (Fig. 6B), and concentration ratios of hypodermis to the dermis were measured to be 10:1 or greater. A similar pattern is shown in Figure 6C (*14*), where an injection drug was also dyed red and showed the telltale fracturing between fat cells to accommodate and drain excess fluid.

### Uncertainty of Equilibrium Concentration Between Subcutaneous Tissue and Dermis

The visual evidence in Figure 6 suggesting that the major portion of the infiltrated radiopharmaceutical remains in the subcutaneous tissue, with limited intrusion into the dermis, was independently confirmed with preliminary ^18^F-FDG experiments in euthanized piglets. The dermis is a largely dense connective tissue, consisting primarily of collagen and elastin surrounded by glycosaminoglycans (highly polar water-binding molecules) that enable collagen fibers to retain water; this water is not freely exchangeable. For purposes of our dose calculations, hypodermis-to-dermis radioactivity concentration ratios of 10:1, 5:1, and 2:1 were simulated, but the results from the 10:1 simulations are likely closest to reality.

### Prior Infiltration Dosimetry Estimates

Several methods for dose estimation of the skin from infiltration events have been reported in the literature. VARSKIN 6.1 (16), a Nuclear Regulatory Commission–sourced Monte Carlo computer code for calculation of superficial skin dose in the case of external skin contamination, has been used in several recent publications. However, unmodified, this model is not appropriate for shallow-dose exposure from activity in underlying tissues. Other published approaches use MIRD methods, or simplified Monte Carlo geometries assuming skin to be a uniform homogeneous tissue, which are inappropriate for a risk assessment in which different skin anatomies have different radiation sensitivities. Osborne et al. (4) present the most complete data on measured clearance rates from infiltration and calculate shallow-dose estimates based on the assumption of uniform distribution of activity into both the dermis and the subcutaneous tissue (equal concentrations), intimate with the sensitive epidermis. This treatment, however, does not account for the β-shielding of the dermis presented in this work, which significantly reduces the shallow dose to the epidermis from β-emissions.

### Radiosensitivity of Skin Subanatomies

Formulated in 1906, the law of Bergonie and Tribondeau states that the radiosensitivity of a biologic tissue is directly proportional to the mitotic activity and inversely proportional to the degree of differentiation of its cells (17). This early concept is foundational to the prevailing understanding of effects from ionizing radiation, and as such, it can be applied to the relevant skin subanatomies in the context of radiopharmaceutical infiltration.

The most proliferative layer of skin is the epidermis, which has a reported mitotic index (fraction of cells undergoing mitosis at a given time) of 0.12–0.14 (18), with complete cell lineage turnover approximately every 40–56 d (19). As a result of the high mitotic rate of the epidermis, deterministic radiation effects can be observed at relatively low doses. Minor and temporary radiation effects (erythema and temporary epilation) can occur at doses of as low as 2 Gy, increasing in severity up to about 8 Gy (20). With acute doses of greater than 10 Gy, more concerning and potentially permanent skin damage can occur, including moist desquamation, ulceration, or scarring, as well as late cosmetic changes, such as dermal thinning and telangiectasia (20). By comparison, subcutaneous fat cells are replaced at a much slower rate, with approximately 10% turnover annually (21). Tissues with particularly slow turnover rates, such as fat and connective tissues, are generally considered to be resistant to radiation. Although data regarding dose–effect relationships are absent from the literature, tolerable subcutaneous doses are likely much higher than thresholds for concerning tissue effects within the dermis and epidermis.

### Infiltration Versus Extravasation

Historically, the terms *infiltration* and *extravasation* have been used interchangeably in the nuclear medicine literature. Clinically, extravasation has a specific definition and refers to injectates that are vesicants—chemicals that are irritants capable of causing tissue damage. In context, this has typically meant that they may be capable of causing blistering, tissue sloughing, or necrosis. The results of this study suggest that in PET imaging we do not approach skin doses in sensitive tissues that would cause such symptoms, nor have they been reported in the literature. For these reasons, in this article, the term *infiltration* is specifically and intentionally used.

### Implications of Results

The lack of clinically significant infiltration events in the 1,000 patients studied, as discerned by quantitative measurement rather than visually based supposition, suggests that a more meaningful definition of infiltration events, as relates to risk assessment for patients, is necessary. With zero patients having more than 1% of the injected activity found at the injection site, none of the 3 adverse event categories introduced at the beginning of the article (tissue damage, diminished diagnostic image quality, or quantitative accuracy) were identified. That is not to say significant infiltrations do not occur. Instances have clearly been reported in the PET literature for the latter 2 categories (although not for the first), and the field at large must be vigilant. With that said, our data indicate a high level of quality in the administration of radiopharmaceuticals in PET practice.

Of particular interest from the Monte Carlo results was the importance of the ratio of activity in the subcutaneous tissue to the dermis, its impact on dose to the epidermis, and the importance of the β-energy of the radionuclide. Using the data and assumptions from this work, the risk of significant tissue injury from diagnostic PET agents appears negligible, as is consistent with both clinical experience and the literature. More interesting, however, are the implications of this work when extrapolated to therapeutic radionuclides and significantly higher injected activities. Importantly, in a review of reported adverse skin events from extravasated therapy administrations (1), most were with ^90^Y, which has an average β-energy of close to 1 MeV and a mean range of 2.4 mm, which is much larger than dermal thickness. The small number of literature reports of ^177^Lu extravasations, with a mean β-range of 0.23 mm (less than dermal thickness), do not yet indicate any significant tissue injury. There is a clear need to extend our current simulation work into therapeutic radionuclides, with therapeutic levels of activity, and to further investigate experimentally the actual quantitative partition of activity between the subcutaneous tissue and the dermis.

## CONCLUSION

The observation of zero clinically meaningful infiltration events in our 1,000-patient cohort having more than 1% of injected activity at the injection site drives a conclusion, with 95% confidence, that the rate for PET infiltrations of more than 1% of injected activity lies somewhere between 0% and 0.37%, substantially below previously reported rates relying on visual interpretation. Further, it appears that when taking into account actual skin anatomy and geometry, absorbed dose to the epidermis from infiltration events is lower than previously estimated because of effective β-shielding by the dermal layer beneath.

## DISCLOSURE

No potential conflict of interest relevant to this article was reported.

## Notes

KEY POINTS **QUESTION:** What is the relative risk of deterministic skin injury from an infiltrated PET injection, considering both the frequency of injection infiltration and the potential absorbed dose to the skin. **PERTINENT FINDINGS:** When characterized by quantitative measurement of activity at the injection site, clinically significant infiltration events for PET radiopharmaceuticals are dramatically less frequent than previously reported in the literature. Absorbed radiation dose to the radiosensitive epidermis is likely substantially lower than previously reported when actual skin anatomy and dose distribution are accounted for. **IMPLICATIONS FOR PATIENT CARE:** Even the infiltration of an entire clinical PET patient dose will likely not lead to any deterministic skin injury.
